# Challenges and opportunities for inclusive, equitable and accessible school holiday clubs for children with special educational needs and disabilities (SEND)

**DOI:** 10.1186/s12939-025-02607-y

**Published:** 2025-09-29

**Authors:** Lorna Hatch, Laura Tinner, Cecilia Khofi-Szeremley, Florence Darling, Sophie Clohessy, Jessica Tanner, Hannah Robinson, Russell Jago, Carolyn Summerbell, Laura Mazzoli-Smith, Miranda Pallan, Margaret A. Defeyter, Marie Murphy

**Affiliations:** 1 Population Health Sciences, Bristol Medical School, University of Bristol, Bristol, UK; 2 Department of Sport and Exercise Sciences, Durham University, Durham, UK; 3 Fuse: The Centre for Translational Research in Public Health, Newcastle upon Tyne, UK; 4 Department of Applied Health Sciences, University of Birmingham, Birmingham, UK; 5 Department for Social Work, Education and Community Wellbeing, Northumbria University, Newcastle upon Tyne, UK

**Keywords:** Children and young people, Inclusion, SEND, School holiday provision

## Abstract

**Background:**

Children with special educational needs and disabilities (SEND), particularly those from families with low-income, experience inequities across educational and health outcomes. The school holidays are difficult for families with low-income, prompting UK government programmes including the Holiday Activity and Food (HAF) clubs. Little is known about how inclusive these holiday clubs are for children with SEND, despite this being a group who may particularly benefit. This study is embedded within a wider project on the HAF programme to explore the challenges and opportunities for inclusive and accessible holiday club provision and provides recommendations for the HAF Toolkit.

**Methods:**

Participant experiences were captured using two qualitative methods: 1) interviews with holiday programme delivery staff and parents of attendees (staff n=28, parents n=10); 2) focus group discussions at creative workshops with parents whose children are eligible for the holiday programme but do not attend (n=22). The Framework Method and Reflexive Thematic Analysis were used.

**Methods:**

Participant experiences were captured using two qualitative methods: (1) interviews with holiday programme delivery staff and parents of attendees (staff *n* = 28, parents *n* = 10); (2) focus group discussions at creative workshops with parents whose children are eligible for the holiday programme but do not attend (*n* = 22). The Framework Method and Reflexive Thematic Analysis were used.

**Results:**

Findings reveal challenges and opportunities around accessing and experiencing the holiday clubs for children with SEND. Access subthemes included: lack of clarity in advertising whether clubs welcome children with SEND; frequent non-disclosure from parents of their child’s needs; accessible transportation; and additional resources needed for SEND provision. Experience subthemes included: food provision for children with SEND; training and staffing that covers the range of needs; and the experiences of children within mainstream provision versus specialist providers of SEND clubs. All participant groups illuminated areas where holiday clubs could be improved to ensure an enjoyable and equitable experience for children with SEND. However, wider debates around ableism and the challenges children with SEND face in society broadly were also illustrated in data. Further, the current economic context and the additional resources needed to support inclusive holiday club provision underpinned much of the data. Opportunities were highlighted such as parent volunteers and external investment, that could maximise the potential of the current government funding.

**Conclusions:**

Our findings highlight issues in access and experience of holiday clubs for children with SEND and provide potential avenues for promoting inclusivity, including how adaptations to the Toolkit could specifically improve HAF. There are considerable challenges to achieving inclusive holiday clubs (financial or otherwise) but if we are to reduce inequities, addressing these should be a public health priority.

**Supplementary Information:**

The online version contains supplementary material available at 10.1186/s12939-025-02607-y.

## Background

For families with a low household income, the school holidays can bring elevated financial pressure, parental stress, food insecurity, social isolation and limited participation in enriching activities [1–4]. Out-of-school periods may be particularly challenging for children with Special Educational Needs and Disabilities (SEND) and their families. The UK Department for Education define a child as having SEND if they “have a learning difficulty or disability which calls for special educational provision to be made for them” [5]. The term SEND covers a wide range of needs which are commonly grouped into four main categories: communication and interaction; cognition and learning; social, emotional and mental health; sensory and/or physical needs [5]. Around 1.7 million children in England receive support for SEND (18.4% of total pupils) with speech, language and communication needs being most prevalent [6, 7]. There remains a diversity of language in this field, so while we have attempted to consistently use ‘SEND’ as our chosen terminology, there is a body of relevant work we draw upon within disability studies, for example, that means we have occasionally used terms such as ableism or disability where appropriate.

There is evidence of inequities in a range of physical and mental health outcomes between children with SEND and those without [8–13], as well as inequities relating to educational attainment. On average, children with SEND experience higher rates of absence [14], exclusion and suspension [15], lower educational attainment during primary and secondary school, and are less likely to go onto higher education compared to their peers without SEND [16–18]. Additionally, children with SEND tend to participate in fewer social and leisure activities [19, 20]. Children and young people with SEND, and their families, often experience the negative effects of stigma and discrimination in the form of bullying, differential treatment by staff, unwanted attention (e.g. staring) and negative reactions to a diagnosis or disability (e.g. complaints or comments about the child’s behaviour) [7, 21, 22]. Drawing upon debates surrounding the medical and social models of disability [23], these inequities partially stem from societal norms and structural ableism [24]. These structural factors restrict the lives of those with SEND, which frames society as disabling as opposed to the individual as disabled (social model). However, there are also real and embodied challenges that many people with SEND face, compared to people without, that may lead to health inequities (medical model) [23, 24].

There is a relationship between SEND and disadvantaged family backgrounds, with children with SEND being more likely to experience socioeconomic disadvantages such as financial hardship. This is particularly the case for children with learning, behaviour or speech difficulties [25]. A child with SEND is also almost twice as likely to be in receipt of benefits-related free school meals (FSM) than a child without SEND [6]. Inequity is intensified for children at this intersection of disability and poverty. For instance, children with SEND from disadvantaged socioeconomic backgrounds are more likely to report inadequate in-school support compared with their more affluent peers with SEND [21]. Further, there is unequal access to appropriate learning support such as securing prompt disability diagnoses and financing private assessments and tuition [21]. There is also evidence that area level disadvantage shapes educational outcomes and experiences for children with SEND [26, 27]. Outside of school, there is some indication that children with SEND and their families feel particularly isolated during the school holidays and would like access to holiday clubs that allow participation in the same enriching activities as children without SEND [28, 29]. Such activities could increase the self-esteem and confidence of the children and provide a much-needed short break for parents. However, finding affordable and suitable holiday activities remains a challenge for these families [30], despite social inclusion in leisure activities for children with SEND having been a national priority in England [31]. Therefore, the difficulties that low-income families face during the school holidays may be amplified if they have a child with SEND, resulting in an exacerbation of inequities. The limited empirical evidence within this area provides a strong rationale to examine how current holiday programmes could be improved to reduce inequities during the school holidays. In response to the additional pressures low-income families face during school holidays, the UK government developed the Holiday Activities and Food (HAF) programme to provide free healthy food and enriching activities for school aged children, primarily for those who receive benefits-related free school meals [32]. Community groups, schools and private organisations can apply to become a ‘HAF Provider’ and deliver a programme during school holidays that includes food and enriching activities. A small proportion of these are ‘specialist providers’ that provide holiday clubs exclusively for children with SEND. These are unevenly distributed across the country and most HAF clubs are delivered as ‘mainstream provision’. HAF programme evaluations estimate that around a quarter of attendees have SEND [33, 34] and they mostly attend ‘mainstream’ clubs [35]. Given that the proportion of children in England with SEND stands at 18.4% [6], there is potentially a higher proportion than the national average attending HAF clubs, which demonstrates the importance of foregrounding their needs.

This paper, which focuses on SEND children and their families, stems from a wider project that explores the HAF programme more broadly. There have been a few HAF programme evaluations that report several positive outcomes for children, young people, and their families [33–37]. For example, an evaluation of the HAF programme in 2021 revealed that HAF programme attendees were significantly more likely to be physically active, participate in outdoor and indoor sports and feel that they had eaten healthy foods over summer than non-HAF programme attendees [34]. Additionally, it has been reported that the HAF programme can increase children’s confidence [35], prevent social isolation and keep children from participating in anti-social behaviour [36]. Furthermore, parents/carers of HAF programme attendees report that the free activities and food bring financial relief [34], and that the provision allows them to work over the school holidays [37]. However, there is sparse evidence for how equitable these outcomes are. There are several challenges with the delivery of the HAF programme, including: its ability to reach the children who may most benefit, a significant number of ‘no-shows’ and, of most relevance to this paper, the delivery of holiday clubs that are suitable for children with SEND [33, 34]. There is acknowledgement among those delivering HAF that children with SEND are likely to be experiencing inequities in access to and their experience of holiday clubs, with parents raising concerns over a lack of clubs that can meet their child’s needs [33]. In response, a Toolkit was developed by the Council for Disabled Children (CDC) intended to equip Local Authority (LA) holiday club coordinators and holiday club providers to support involvement of children with SEND in holiday club opportunities in their local area. The Toolkit reports barriers to participation and offers information and tools to support delivery. For example, this includes a ‘SEND Checklist’ for holiday club coordinators and providers, as well as ‘example booking form questions that promote inclusion’. Yet critical gaps remain around the challenges and opportunities for enhancing the equity of holiday club provision, from the perspectives of those who deliver holiday clubs, the families who attend them and those who are eligible but do not attend.

Data from our participant samples highlighted issues around inclusivity and accessibility specifically in relation to SEND, that warranted distinct attention. Therefore, the aim of this paper was to explore the challenges and opportunities for the promotion of inclusive and accessible school holiday provision, such as the HAF programme, for children with SEND who are experiencing poverty, from the perspectives of parents and practitioners.

## Methods

### Study design

This is a multi-method qualitative study that used interviews and focus group discussions.

### Sampling and recruitment

#### Interviews

We recruited staff who deliver the HAF programme (*n* = 28). The participant groups were: local authority (LA); political leads (*n* = 5); HAF programme leads (*n* = 11); and HAF providers (*n* = 12). These individuals were eligible for participation if they were involved in delivery of the programme in their respective regions. We also recruited parents (*n* = 10) of children who use the programme to participate in an interview. They were eligible if their child/ren had attended at least one session. We used purposive sampling, taking a maximum variation sampling approach, using existing contacts, internet searches, and through recommendations of other participants. This yielded a sample that included participants from across Southwest, Midlands and Northeast of England, and the clubs recruited from varied in the geography of their area, size, and activities provided and meant participants varied in their experiences and perspectives. Interviews were chosen for the HAF delivery staff and parents who attend to benefit from more in-depth and one-to-one conversations. Some of these participants were already known to us (e.g., a couple of the LA political leads) and some were accessed through our networks (e.g., LA HAF delivery staff recruited through existing LA contacts), however most participants were unknown to the research team. Moreover, recruitment was monitored to ensure diversity in demographic characteristics, geographical spread, and HAF delivery model.

#### Focus groups with parents at family creative workshops

We recruited children and their families to seven creative workshops (*n* = 37 children and *n* = 22 parents or carers) across the study sites in the Northeast, Southwest and Midlands of England. Participants were purposely recruited through organisations with established relationships with low-income families such as schools, alternative provision providers, family support services and voluntary and community organisations. The only criterion was that families were eligible for their children to have free school meals (and therefore eligible for HAF) but were not attending HAF (or had stopped attending). Most of the parent sample had at least one child with SEND, which was disclosed to the researchers during conversation as we did not collect demographic data on SEND diagnosis. Workshops were delivered in a space within these organisations that was familiar, safe and comfortable for the adults and children taking part. This method was chosen to complement the interviews as this group of participants were not already known to us through HAF networks and we needed to work closely with community partner organisations to recruit these families. We therefore chose to collect data through focus group discussions with parents who were already attending an in-person workshop with their children, held at a community setting that they had previously used, rather than ask them to engage in an interview in addition to the workshop.

The rationale for our choice of participant groups stems from our objective to understand how the programme can be improved to be more inclusive and accessible. It is crucial to speak with those who deliver HAF to understand the operational challenges and improvements that can realistically be made. It is also necessary to gather data from families whose children do attend the programme as well as families who are not currently attending to provide a full picture of the barriers to access and how the experience at the holiday clubs could be improved.

### Data collection

Online interviews were conducted with LA political leads, HAF programme leads, HAF providers, and parents of attendees of the HAF programme. Interviews lasted 30–60 min, with most being around 45 min. Semi-structured topic guides (Additional files 1–6), with open-ended questions and prompts, were developed for each participant group. HAF staff were asked about how they deliver the programme, how well they think the programme reaches and engages with families, how they evaluate the programme and general challenges and successes. Parents were asked about their engagement with HAF, the impact on their child’s life, positives and negatives of the programme and any suggested changes for improvement.

There were several activities within the full-day creative workshops delivered in community settings, but in this present paper we focus only on the data gathered from the parent focus groups. The methodological approach and findings from the child-focused and whole family activities within the creative workshops are reported in detail in two forthcoming papers (39). In the focus groups we asked few direct questions about inclusivity around SEND, yet this dominated many conversations. Broadly, topics included what families are currently doing in the school holidays, what they would like to be doing and what the barriers and enablers are to them having a positive experience.

### Analysis

The Framework Method [40], which includes seven stages (Additional file 7) [40], was used to organise, code and map the data. Members of the research team engaged in the framework method independently, following the stages to code data into a framework matrix. This data management was conducted using NVivo version 12 software (QSR International). The Framework Method provided a structured way for us to organise the data, which is suitable for an applied approach such as this, where we could allocate data to our pre-determined objectives and have a matrix of the different participant groups. The framework matrix developed through this process was used as an aid to our data analysis, which was conducted as a team using reflexive thematic analysis [41].

From the initial reflexive thematic data analysis, data on the topic of inclusivity of children with SEND arose inductively; there was a large quantity of data – with most participants discussing it – and it related to all elements of HAF programme delivery. These data required further exploration and could not be captured accurately as a single theme in our overall findings paper. Moreover, as an active part of our reflexive practice, we reflected on and acknowledged our positions of privilege compared to our participants [42]. This reflexivity led to our agreement that it would be an equity issue to not report these data related to SEND provision in the comprehensive manner that they require. While interview and focus group data were initially analysed separately, upon realising the pattern of data related to SEND inclusivity, we revisited and analysed all the data together and held meetings to discuss with this renewed focus. We created a new Framework matrix and the six-phase reflexive thematic analysis was followed, whereby the team generated initial codes, searched for themes, reviewed themes, and defined and named themes. To conduct the additional analysis required, the team met regularly to discuss the data relating to inclusivity of children with SEND. Throughout meetings we addressed conflicts and practiced reflexivity through acknowledging our positionalities and discussing how these may impact our interpretations. Of relevance here, none of the analysis team were currently experiencing poverty, had sent their child to a HAF club or had SEND, meaning we all occupied an ‘outsider’ status [43] but also a closer proximity to power. Through inductive coding, two main themes of access and experience were identified, with considerable challenges but also good practices being discussed within these areas. This led to the development of the main themes and sub-themes for this paper.

### Ethics

Ethical approval for this study was granted by Faculty of Health Sciences Ethics Committee at the University of Bristol (Ref: 13642) and Durham University’s Ethics Committee (Ref: EDU-2023-12-14T09) owing to the different site leads for the different parts of the project. Informed consent was collected for all participants. Confidentiality and anonymity were ensured by replacing names with participant codes and removing potentially identifying information from the transcripts. Data were stored securely on an electronic server, with access restricted to the researchers on the team.

## Results

### Participant characteristics

LA political leads (*n* = 5; 5 female), HAF programme leads (*n* = 11; 7 female, 4 male), holiday club providers (*n* = 12; 8 female, 4 male) and parents/carers (*n* = 10; 7 female, 3 male) were interviewed. Four providers (25%) were specialist SEND provision and the remainder mainstream provision. Twenty-two parents/carers (95% female) took part in in-person focus groups. As we recruited from a population of participants whose children were eligible for free school meals (i.e., we did not target families with SEND), and the challenges of SEND provision came up organically, we did not collect demographic data on SEND diagnoses of the families.

### Themes

We have separated our findings into two overarching themes: *Accessing holiday clubs* and *Experience of holiday clubs*. Under each we present several subthemes which explore the challenges and opportunities for accessible and inclusive holiday club provision for children with SEND.

### Accessing holiday clubs

#### Advertising the programme

One challenge relating to children with SEND being able to access a holiday club was the marketing of the programme. While this appeared to be a broader difficulty with HAF, within the context of SEND there was a need for detailed and accurate adverts that let families know whether the club will be suitable for their children, given their needs: “*[we need to] help parents understand*,* you know*,* ‘my child has low needs so this provision might be suitable’*,* ‘my child has high needs*,* I need to look at this end of the website’*,* so [there’s] some work there.”* (HAF Lead 5). The onus is currently on families to investigate either on the day or contact ahead of time, whether the holiday club is appropriate for their child.

There is potential here to promote inclusivity and accessibility of the HAF programme to children with SEND; something that could be achieved with more transparent communications and discussions with families. Several HAF Provider participants had ideas or were trialling systems to promote greater access: “*We’ve actually*,* um*,* developed a slightly different version of the [branding] – so that*,* when families are looking for activities*,* they’ll know that those are activities specifically for SEND*” (HAF Lead 5). One parent had taken the initiative to develop a grading system in collaboration with the local authority that mapped out the different possible needs that children may have, for clubs to assign themselves a grade based on their facilities and resources: “*So I’ve sat down with the local authority and introduced a banding system in [area] where there’s four different levels of accessibility. So one is like your mainstream*,* two was your inclusive*,* three is your SEN session and that is more where it’s targeted for families with special educational needs*,* but where the parents are meant to stay and band four is an actual staffed session where the parents would be able to leave their children.*” (Parent 5). This parent was awaiting more widespread uptake of the system, but we interpreted this as one promising avenue to allow holiday clubs to advertise their provision and for parents to confidently attend clubs they knew would be suitable for their child.

#### Disclosure of SEND

From the perspective of the HAF delivery staff, participants regularly experienced parents not disclosing that their child had SEND, leading to the child either being turned away from mainstream HAF clubs upon arrival, or having a negative experience, due the clubs being unsuitable for the child: “*we had children turning up in the summer to HAF programmes but parents not declaring – or telling us – what their children’s additional needs were. Um*,* so*,* their experience was not as they should do”* (HAF Lead 2). We interpreted this issue to be complex and occurring for different possible reasons, including: [1] a lack of official diagnosis or clear understanding of the child’s need [2], stigma around the child’s condition preventing disclosure [3], the lack of any activities for the child to do during the holidays driving parents not to disclose SEND and hope the club would accommodate on the day, and [4] advertising of the club suggesting inclusivity, meaning parents did not feel they had to disclose their child’s needs. Aside from improving the inclusivity of the HAF programme overall as well as the transparency around what each club offers, the main suggestion to address the challenges of non-disclosure was around having more honest conversations with families and spending time to build those relationships.

#### Getting to the club

Transportation was a key issue for families with a child with SEND, which was a barrier to access noted by programme delivery staff, parents of children who attend HAF and parents of children who do not. For instance, one provider of a specialist SEND club reported: *“families would*,* would phone and say – I can’t come today because I don’t have transport*,* or I don’t want to come because it’s two bus journeys across the city”* (Provider 11). There are further complexities for many families aside from the distance and cost of travel (which is a challenge for all children hoping to attend holiday provision). This provider highlighted that issues such as these are further exaggerated when the children have SEND in terms of the extra travel time needed for those with physical impairments, but also public transport not being suitable for children with neurodivergence. Several families spoke of not having their own car and public transport was not possible for their children with SEND: “*My daughter don’t like public transport*,* she kicks off. On her EHCP [Education*,* Health and Care Plan]*,* it says she has to have 2:1 [ratio of adults to child] … but I’m a single parent of four kids*,* I ain’t got 2:1”* (Parent 31). The main opportunity for these families to attend holiday provision, was for private accessible transport organised by the provider, which some did arrange, but this has cost implications and impacts on the number of places the clubs can offer.

#### Finance, resourcing and operations

The major barrier, unsurprisingly, which underpins the previous subthemes, is the additional resource it takes to deliver a holiday club that is accessible and inclusive of children with SEND. This relates to both specialist provision: “*there should be a special provision for SEND and neuro[diverse] children. But there just wasn’t the capacity to deliver that within the funding and within the resources*” (Provider 10), as well as improving the inclusivity of mainstream clubs by resourcing more staff, accessible facilities and transportation.

To address this issue, the most common suggestion participants raised was around ringfencing funds from the government funding they received to be used to develop their SEND offer to children: *“[we have] dedicated an element of the funding since the summer – and it works really well… dedicated an element of the funding for providers who want to deliver activities specifically for children and young people with SEND”* (HAF Lead 6). Given the cuts to public funds and that the holiday clubs are already finding it a huge challenge to reach thousands of children experiencing poverty with their current funding, some participants suggested collaborating with private enterprises and charities or seeking funding from elsewhere to try and achieve their aims: “*we had a grant from the inclusion service*,* which meant we could yes*,* support that specific child [with one-to-one support]*,* which was amazing. Like we’ve never been able to do that”* (Provider 2).

Several participants mentioned that accessibility of some clubs was very difficult to overcome without significant additional funds, particularly if the issues were around the venue of the club or the number of staff, due to capacity issues in the community voluntary sector already: *“not all of our clubs are massively accessible because they’re really old buildings. Children with visual impairments*,* you know*,* it might not work for them”* (Provider 6). One parent suggested extending capacity through providing volunteering and basic wage ‘upskilling’ opportunities: “A*sking if any students want to volunteer…student occupational therapists*,* speech and language therapists*,* those that are going onto specialist nursing. I mean*,* it doesn’t all have to be money. Some people are desperate for that experience”* (Parent 5).

### Experience of holiday clubs

#### Food provision

Several HAF delivery staff noted the difficulty in providing food suitable for children with SEND (many of whom have individual and specialised diets) within the context of holiday club provision, as there are certain ‘healthy food’ criteria (in the UK termed ‘school food standards’ [44]) that must be met to be able to be a HAF club. The result of having to cater to several children and meet the school food standards on a limited budget, regularly meant that children with SEND missed out on the food provision within HAF: *“We had no choice with [HAF club] because they couldn’t meet his needs. We had to take a packed lunch.”* (Parent 5). One example where a club was particularly successful with food provision was where they worked with the chefs at a special school: “*the school kind of sent through their menu of this is what we provide at school so she was kind of like taking what they are used to*,* um*,* and made meals around that”* (Parent 2). This model was only possible as it was a small club that was designed for children who attended the special school already.

HAF also presents an opportunity to support children with SEND to try new foods, which not only has the benefit to the child through being able to eat a healthy meal, but through improving confidence and independence, broadening their experience. One way of providing the chance to try new foods for children with SEND is through getting them involved in food preparation: *“He finds food preparation quite regulating. He zones out and he’s quite calm and he’s happy. So*,* the food tech team are absolutely brilliant. We had a chat with some kids yesterday*,* actually*,* and they said similar things they really liked getting involved in cooking the food or just preparing the food”* (Parent 5). This was not only something mentioned in regard to children with SEND, but to all children. HAF delivery staff noted that limited knowledge of food and hesitancy to try food was a widespread issue across the HAF programme that risks food waste and children leaving the programme having not eaten anything. Therefore, as well as specific benefit for children with SEND, integrating more conversations about food and getting the children involved in preparation may have wider benefit to all attending children.

#### Training and staffing

Specialist training of staff to support working with children with SEND was lacking across all mainstream providers, as was having the numbers of staff needed to deliver SEND provision: “*Providers have come to us and like*,* you know*,* “we don’t have the ratios properly or we don’t have the expertise*” (HAF Lead 5). This automatically excludes some children with SEND from attending a club, for instance, if there are not enough trained staff: “*it*’*s through having enough staff*,* enough training and enough resources and understanding. There’s a lot of associations I find*,* they ain’t got a clue about it*,* they have simple training and it’s like*,* nah you ain’t got a clue”* (Parent 30). This issue also impacts on children’s enjoyment of the club, if they are not properly supported, with some parents highlighting instances where they had to collect their child, or their child saying they did not want to go back to the club again.

The obvious need here is around increasing and enhancing training and capacity of HAF club staff to support children with SEND. Given that most delivery staff we interviewed noted that most mainstream clubs will have some children who attend with some level of additional needs, it appears that introducing this kind of training should be as standard. However, increasing training would not necessarily get around the broader issue of very limited staff capacity within the community voluntary sector, which makes it difficult to deliver the clubs as it is. Increasing the numbers of staff so that children with SEND are safe and have a good experience, felt like an impossible task in this context: *“I don’t think we quite appreciated how much staffing was needed for the safety element of it. The level of behaviour challenges in that community was quite complex. There was a lot of… ADHD*,* like low level sort of learning disabilities and kind of a lot of just behaviour problems”* (Provider 12). An opportunity to address capacity and trained staff issues was interpreted from the parent data. Several parents of children with SEND said they did not feel comfortable leaving their child at a holiday club alone, with a few volunteering at clubs so they could be present (as most clubs do not allow parents to stay). If parents who need to stay with their child could be welcomed to be active volunteers at holiday clubs, their skills and experience in interacting with children with SEND could be utilised and support other staff members, at relatively little cost to the programme: “T*here was a group of kids that kind of navigated towards me that were those that I could pick out as neurodiverse*,* but anyone without my background wouldn’t have picked them out”* (Parent 5).

#### Mainstream provision

Most of the challenges that participants described in relation to inclusivity of children with SEND were within the context of ‘mainstream’ provision, or HAF clubs that are not specifically designed for children with SEND. The specific difficulties are outlined in the previous themes, related to staffing and training, food provision and disclosure. What our participants highlighted here was a broader question around the extent to which mainstream provision should be inclusive of all children, given the increasing proportion of children with SEND and the diverse level of need. On the whole, HAF delivery participants found this to be a priority, but were often restricted by budget and lack of an accurate understanding of what was needed to enhance inclusivity: “*there’s a huge*,* huge amount of people who need that additional support so rather than excluding them or having specialist provision they still want to take part in things with friends*,* they don’t want to feel segregated and that’s some of the things which we’ve learnt whilst talking to our providers… is*,* it’s not cheap erm but err is highly valued”* (HAF Lead 11).

#### Special schools

Special schools appeared to be a great support and signposting resource that provided specialist HAF clubs for small groups of children with the greatest level of additional needs: “*this year we worked really hard*,* we got four special schools to deliver the programme to their own children which worked really well”* (HAF Lead 2). This included providing venues, food provision, staff and suggesting activities. The greatest challenge was around children not necessarily wanting to spend their holidays in school: “*school is quite triggering for him*” (Parent 5). It also risks the segregation discussed in the previous theme and has the potential to discourage mainstream clubs from enhancing inclusivity, as they think that children with SEND are catered for elsewhere. This again draws out a conflict in that some children will need significant support, but taking a social lens to disability highlights how it is wider society that is, to some extent, disabling these children. Therefore, it is not only about additional resources but also shifting norms and perspectives regarding the diverse needs of children with SEND. Providing a holiday club that all children can attend is the ideal scenario to break down disabling structures and discourse. However, in a purely pragmatic sense that has been explored by the HAF delivery staff, the programme does not yet have widespread inclusive structures to ensure safe and enjoyable attendance of HAF for some children. This does not necessarily identify HAF as a non-inclusive programme but instead is reflective of a wider disabling society.

## Discussion

Our study reflects the challenges and opportunities for inclusive and accessible holiday food and activity clubs for children with SEND. We found there to be significant challenges to improving the access to holiday clubs for this group of children, for instance: the lack of clarity in advertising whether clubs welcome children with SEND; frequent non-disclosure from parents of their child’s needs; accessible transportation; and additional resources needed for SEND provision. There were several themes related to how the holiday clubs can be made a positive experience for children with SEND, including: food provision for children with SEND; training and staffing that covers the range of needs; and the experiences of children within mainstream provision versus specialist providers of SEND clubs. These findings are situated within an evidence base surrounding the HAF programme but also scholarly debates on equitable access and experience of holiday clubs and more broadly public health interventions for individuals with SEND.

We identified similar challenges to those noted in the Toolkit [38] designed to support SEND inclusivity of HAF programmes, such as how programmes are advertised, limited finances and resources and the training of specialist staff [38]. In terms of accessing the clubs, HAF delivery participants in this study highlighted that parents not disclosing that their child had SEND is a common experience. Similarly, Bayes et al. [35] surveyed HAF providers and found that children’s needs and requirements were not always mentioned, which impacted on their ability to include children and also had cost implications. Parents not disclosing the full needs of their child in advance was also highlighted as a barrier to participation for children with SEND by HAF programme leads and providers in the Toolkit [38], which emphasises the importance of booking forms that use supportive language to encourage families to share the right information [38].

There is a body of evidence on SEND disclosure of children, largely in relation to autism [44–47], highlighting the dilemma many parents face when deciding whether to inform holiday club leaders about their child’s needs, which is not always straightforward and risk-free. A core consideration for parents is around stigma, which we know from a vast literature that people with disabilities are at high risk of experiencing [48–50]. This fear of stigma, including the perception that a child will not be able to attend if their additional needs are mentioned, may be in part driving low disclosure of SEND within the HAF programme; inadvertently leading to several of the issues in access and experience that we have identified. A systematic review revealed that despite initial fear of disclosure adolescents and adults often had positive outcomes as a result of disclosing their autism diagnosis [48]. The evidence base is far more limited related to disclosure among younger children, we therefore must situate our findings within research on adolescents and adults, which is problematic. However, our HAF delivery participants explicitly highlighted the challenges they face in relation to the range of additional needs among children and how low disclosure adds greater complexity and reduces their ability to be inclusive, which chimes with the issues highlighted within adolescent and adult literature. What we argue is that parents and families may need support and guidance to feel confident to disclose their child’s needs, with assurances that disclosure will not lead to exclusion of their child.

The challenges highlighted by parents of children with SEND regarding getting to the holiday clubs represents a central point when thinking about accessibility and inclusivity. Society (including transport and physical spaces) outside of the HAF clubs is not easy to navigate for families with a child with SEND, within the context of the social model of disability that highlights it is the structures around us that are disabling and need changing, rather than the individuals [52]. Therefore, creating a club that is truly accessible and inclusive of children with SEND means communicating with families to understand all the barriers preventing inclusion. It is not enough simply to provide accurate and transparent advertising of the level of inclusivity of the holiday club. This idea aligns with the concept of ‘poverty proofing’, which we have explored in relation to how families on low-income can access HAF (Hatch et al., in final stages of review at BMC Public Health), in that there needs to be a consideration of all the additional barriers, costs and unintentional stigmas that can arise from otherwise free initiatives [53]. We highlight a similar line of thinking here in that inclusivity in relation to SEND is not just about the holiday club itself, but also consideration of the wider exclusions experienced in terms of embodied capital, social norms and ableist structures that constrain individuals’ engagement with the holiday clubs. We provided the example of transport within our data that displays how SEND inclusivity extends beyond the holiday club.

In relation to experience of the holiday clubs for children and young people with SEND, there was a pattern of data that chimed with wider debates around the balance of investing in and promoting specialist provision versus enhancing the inclusivity of mainstream settings. One evaluation of the HAF programme found a similar tension in that some families liked having the option of specialist SEND provision for their child and wanted that offer to be protected financially, but others saw the benefit of their child going to mainstream clubs and did not necessarily want their child to be segregated [33]. There is a parallel literature within the school context, propelled by The Warnock Report in 1978 [54], which sowed the seeds for a move away from special schools towards inclusion within mainstream education. Shah [55] found that children with SEND have preferences for both mainstream and special educational settings, illuminating that they are not a homogeneous group. There is also no clear steer on whether children get better outcomes in one setting over another [56]. Several recent studies have built a line of argument to suggest that a model of special and mainstream school partnership may be the best approach for inclusivity [56, 57]. Our data and others related to HAF are beginning to align with the school context, although greater exploration of the dynamic between specialist HAF clubs and mainstream provision is needed to understand whether a partnership approach would be a promising route to a more inclusive holiday club programme.

The reality of an under-funded public sector and the economic challenges of working to deliver community-based initiatives cannot be underestimated. The need for additional funding and resourcing to ensure inclusivity and accessibility for children with SEND was mentioned by several participants. However, there were also a few suggestions around how resourcing could be more efficiently used within the HAF programme, to enhance the offering to SEND children and promote inclusivity. Engaging parent volunteers, work experience programmes or additional funding from charities or businesses (as breakfast clubs in the UK did from Kellogg’s [58]) could go some way to addressing gaps in SEND holiday club provision. What is important is that initiatives think creatively and keep inclusivity at the core of what they do. Asset-based approaches can be useful here in assessing what skills and resources are already present within the community and how they can be better utilised [59].

### Strengths and limitations

While we collected demographic data for the interview participants, there are limitations around certain characteristics. Our convenience sampling for HAF delivery staff means we may have missed local authority locations facing the greatest challenge. Further, several participants (namely parents) described the additional needs of their child, but they had not yet received any formal diagnosis or declaration of SEND. Additionally, an open text box for ethnicity meant that we cannot accurately display the ethnic diversity in the sample as several people listed nationality or left it blank. Further, we did not go into the study with an intention to uncover challenges and opportunities specifically related to children with SEND, but we explored broadly how inclusive and accessible HAF is as part of a broader examination of the holiday club programme. Despite not asking direct questions about this topic, discussions related to SEND arose naturally and therefore warranted dedicated exploration, but for this reason we do not have detailed participant data about SEND and could have asked further questions on this topic to illicit further useful data. We hope to delve deeper into the experiences of children with SEND related to holiday club and public health interventions in future studies.

This project also highlighted to us a gap within our skillset and knowledge around SEND as public health researchers, and frameworks or examples of how it is achieved in practice. While we see it as a strength of this study that we used approaches that made it possible for children with SEND to take part (i.e., creative activities, adapted topic guides, one-to-one interviews with parents there to support) we felt as a team there was real need for researcher training, which mirrored the experiences of the HAF delivery staff. We also found that resources to support the research process for people with SEND was lacking (e.g., wider range of tools for informed assent/consent and alternative data collection procedures). We would be supportive of further training and resources, potentially drawing upon organisations outside of academia working in this space, that would mean researchers are fully equipped to include and support children and families with SEND in projects in a meaningful and equitable way.

### Policy and practice recommendations

Table 1 summarises the challenges and opportunities identified within the present study for inclusive holiday provision for children with SEND, with reference where applicable to the HAF programme Toolkit. We continue to work with the Department for Education to establish how our recommendations (Table 1) can inform future policy and practice around inclusion holiday club provision.

**Table 1:** Challenges and opportunities for inclusive holiday provision for children with SEND, as identified from this study, alongside recommendations for updates to the toolkit based on these findings

**Code/theme**	**Challenges**	**Opportunities**
**Accessing holiday clubs**		
Advertising the programme	• Lack of information on if/how children with SEND (and their siblings) are eligible for the programme and which clubs are accessible to them. • Onus is on families to investigate, either on the day or contact ahead of time, whether the holiday club is appropriate for their child. • At times families are told clubs are accessible to children with SEND but then arrive and it is not suitable.	• Implementation of a grading system to indicate the level of inclusivity and accessibility of each club to families. **Recommendation**: *This resource could be a useful addition to the Toolkit*,* as a comprehensive and standardised method to share club inclusivity information with families.* • Transparent communications and discussions with families. • Value and opportunity of providers linking with SENCOs in local schools to advertise the programme.
Disclosure of SEND	• Families not declaring their child’s needs to clubs due to: a lack of official diagnosis or clear understanding of the child’s need; stigma around the child’s condition preventing disclosure; the lack of any activities for the child to do during the holidays driving parents not to disclose SEND and hope the club would accommodate on the day; and advertising of the club suggesting inclusivity, meaning parents did not feel they had to disclose their child’s needs. • Some ‘mainstream’ clubs declare to be inclusive and accessible, but then not actually equipped when families arrive.	• Working closely with families to understand children’s needs and what would make the holiday club activities inclusive. • Communications/booking forms that use supportive language to enable families to feel confident to disclose their child’s needs, with assurances that disclosure will not lead to exclusion.
Getting to the club	• Transport is a particular challenge for the clubs more broadly, but especially for children with SEND who may not be able to use public transport or have access to a vehicle.	• Working with families to understand the access issues related to physically getting to the club. • Clubs providing transport to enable children with SEND to attend.
Finance, resourcing and operations	• Restricted funding for the programme as a whole and limited funding for provision for children with SEND, which costs more.	• Ringfencing programme funds to develop the SEND offer. • Allocating additional funds for providers who make SEND adjustments. • Collaborate with organisations with experience and training in working with children with SEND. • Programme and club staff seeking additional funding, such as from private and third sector organisations, to support provision for children with SEND. **Recommendation**: *Approaches for putting this in action with examples or case studies would be a helpful addition to the Toolkit.* • Offering volunteering, work experience and basic wage upskilling opportunities to increase club staff capacity, such as for student occupational therapists, speech and language therapists or nurses. **Recommendation**: *These could be added to the Toolkit as additional examples to those already mentioned e.g.*,* Young Leaders.*
**Experience of holiday clubs**		
Food provision	• Difficulty providing food suitable for children with SEND within the context of holiday club provision, while also meeting School Food Standards and with a limited budget.	• Working with special schools to develop menus and provide food. • Involving children and families in food preparation. • Providing lots of options. **Recommendation**: *A section on food provision*,* with specific recommendations such as those above*,* would be a valuable addition to the Toolkit.*
Training and staffing	• Limited number of experienced staff to support children with SEND.	• Training as standard for club staff on supporting children with SEND. • Providing opportunity for parents of children with SEND to volunteer at the clubs to support capacity. **Recommendation**: *The Toolkit would benefit from guidance on implementing this in practice.*
‘Mainstream’ provision	• Difficulty for ‘mainstream’ clubs in meeting children’s needs, due to increasing proportion of children with SEND and the diverse level of need. • The aim to include children with SEND, rather than segregate, requires additional resources and training, including in how to support children with SEND without stigmatising.	• Working with children and families to enhance ‘mainstream’ provision to be as inclusive and accessible as possible. Simple changes could support children with SEND and improve the provision for all children. • Use of a framework for engaging with families to understand their child’s level of need and which clubs they could attend. **Recommendation**: *While example methods for collecting this information have been mentioned in the Toolkit (e.g.*,* via phone call)*,* a framework for engaging with families*,* including approaches to take when challenges arise*,* would provide additional support for staff.* • Need for routine training of all staff.
Special Schools	• Children may not want to go to school during holidays, and the environment can be triggering for some children. • Use of special schools could lead to risks of segregation and potential to discourage mainstream clubs from enhancing inclusivity, as they think children with SEND are catered for elsewhere.	• Special schools have accessible facilities and specialist staff meaning better inclusion for those with more complex needs. • Special schools could support ‘mainstream’ provision through signposting families to inclusive clubs, advising on food and activities, and providing food for clubs. School staff could also support delivery at ‘mainstream’ clubs. **Recommendation**: *A future Toolkit could build on its suggestion to link with SEND specialists by providing examples of who and how they might be able to help*,* such as those mentioned here.*

## Conclusion

This study outlines the challenges and opportunities for equitable holiday club provision for children with SEND. Our findings illuminate issues in access and experience of holiday clubs, providing potential avenues to explore for promoting inclusivity. We also highlight salient debates around mainstream versus specialist school and holiday club provision, as well as how best to optimise limited public funding in community settings for inclusivity. We conclude that despite several challenges there are some opportunities to improve the access and experience of HAF for children with SEND. These opportunities may only be realised through strong engagement with families, creative thinking around funding and asset-based thinking. We have presented these opportunities as practical recommendations for the HAF Toolkit for inclusivity [38], which our data suggest will enhance holiday club provision for children with SEND.

## Supplementary information

Supplementary material 1. Topic guides for interviews and focus groups

Supplementary material 2. Topic guides for interviews and focus groups

Supplementary material 3. Topic guides for interviews and focus groups

Supplementary material 4. Topic guides for interviews and focus groups

Supplementary material 5. Topic guides for interviews and focus groups

Supplementary material 6. Topic guides for interviews and focus groups

Supplementary material 7. Analysis plan

## Data Availability

The datasets supporting the conclusions of this article are available in the University of Bristol data archive: https://data.bris.ac.uk/data/.

## Abbreviations

SEND: Special educational needs and disabilities
FSM: Free school meals
HAF: Holiday activities and food
LA: Local authority
